# Combination of Berberine and Evodiamine Alleviates Obesity by Promoting Browning in 3T3-L1 Cells and High-Fat Diet-Induced Mice

**DOI:** 10.3390/ijms26094170

**Published:** 2025-04-28

**Authors:** Huiying Zhang, Peiyu Xiong, Tianyan Zheng, Youfan Hu, Pengmei Guo, Tao Shen, Xin Zhou

**Affiliations:** School of Basic Medicine, Chengdu University of Traditional Chinese Medicine, Chengdu 611137, China; zhanghuiying@stu.cdutcm.edu.cn (H.Z.); xiongpeiyu@stu.cdutcm.edu.cn (P.X.); zhengtianyan@cdutcm.edu.cn (T.Z.); huyoufan@stu.cdutcm.edu.cn (Y.H.); guopengmei@cdutcm.edu.cn (P.G.); st1963@263.net (T.S.)

**Keywords:** berberine, evodiamine, obesity, adipose tissue browning, Fibroblast Growth Factor 21

## Abstract

Traditional Chinese medicine has long acknowledged the therapeutic potential of *Tetradium ruticarpum* (A.Juss.) T.G.Hartley together with *Coptis chinensis* Franch in managing metabolic disorders. However, their combined anti-obesity effects and the underlying mechanisms remain poorly characterized. This study investigates the synergistic anti-obesity effects and mechanisms of a combined berberine and evodiamine treatment (BBE) in high-fat diet (HFD)-induced C57BL/6J mice and 3T3-L1 cells. In vitro, cell viability was evaluated using the Cell Counting Kit-8 (CCK-8), while lipid accumulation was assessed through Oil Red O staining and triglyceride content determination. Molecular docking simulations performed with AutoDockTools 1.5.6 software Vina predicted interactions between BBE and key proteins. The analysis of genes and proteins involved in browning and thermogenesis was conducted using quantitative reverse transcription polymerase chain reaction and Western blotting. In vivo, HFD-induced mice were assessed for serum lipids profiles, glucose, insulin, adipocytokines, fat tissue morphology (Hematoxylin and eosin staining), mitochondrial activity (flow cytometry), and protein expression (immunofluorescence). Molecular docking analysis revealed strong binding affinities between BBE and key target proteins, including UCP1, PGC-1α, PRDM16, CIDEA, FGF21, and FGFR1c. BBE significantly reduced lipid accumulation in 3T3-L1 cells, upregulated the mRNA expression of *Prdm16*, *Cidea*, *Ucp1*, and *Dio2*, elevated UCP1 and PGC-1α protein levels, and activated the FGF21/PGC-1α signaling pathway. In HFD-induced mice, BBE administration led to reduced body weight, smaller adipocyte size, increased adipocyte number, and alleviated hepatic steatosis. Furthermore, it lowered serum total cholesterol (TC), low-density lipoprotein cholesterol (LDL-C), and levels of triglycerides (TG), while simultaneously increasing concentrations of high-density lipoprotein cholesterol (HDL-C). BBE also improved glucose tolerance, reduced fasting insulin levels, and modulated adipocytokine levels (reduced leptin, increased adiponectin), while promoting browning gene and protein expression. Overall, the combination of berberine and evodiamine mitigates obesity by enhancing browning and activating the FGF21/PGC-1α signaling pathway.

## 1. Introduction

As a chronic metabolic condition characterized by the excessive accumulation and dysfunction of adipose tissue, obesity results from a complex interplay of genetic susceptibility, obesogenic environment, and psychosocial factors [1]. The World Health Organization (WHO) 2022 report indicates that over 890 million adults globally are categorized as obese (BMI ≥ 30 kg/m^2^), representing approximately 16% of the global adult population [2]. Moreover, obesity is closely linked to severe conditions, such as diabetes, obstructive sleep apnea, and high-mortality cardiovascular diseases, imposing substantial economic burdens on global healthcare systems. It is projected that by 2030, annual healthcare costs related to obesity and overweight will reach three trillion USD [3]. Therefore, addressing obesity is imperative, not only to alleviate clinical symptoms, such as excessive weight gain, hyperglycemia, hyperlipidemia, and adverse fat distribution, but also to fundamentally investigate the underlying mechanisms, including reduced fat absorption, improved insulin sensitivity, and the modulation of gut microbiota, with enhancing adipose tissue browning being a key approach for the prevention and control of obesity [4].

Adipose tissue browning is primarily characterized by the phenotypic transformation of adipose tissue, specifically the conversion of white adipose tissue (WAT) into a more thermogenic and metabolically active form, which is particularly significant in the amelioration of obesity. A reduction in brown adipose tissue, increased white adipose tissue (WAT) formation, and decreased stimulation of beige adipose tissue can all lead to reduced energy expenditure, thereby promoting the development of obesity. WAT primarily functions to store excess calories and regulate endocrine function and energy homeostasis through the secretion of adipokines, such as adiponectin, leptin, and resistin [5]. The brown adipose tissue (BAT), which is rich in mitochondria, demonstrates an elevated expression of mitochondrial intimal uncoupling protein 1 (UCP1), where UCP1 promotes proton leakage, converting the potential energy from nutrient metabolism into heat rather than storing it in ATP [6]. Beige fat, typically found within WAT, can be transformed into beige adipocytes with characteristics of brown fat cells in response to cold or β-adrenergic signals, a phenomenon known as WAT browning [6,7]. Small lipid droplets, elevated UCP1 expression, and high mitochondrial content are common features of beige and brown adipose tissues, serving as key markers that differentiate them from WAT [8]. Given the strong association between WAT browning and metabolic health, WAT browning has emerged as a crucial target for obesity interventions [4].

Traditional Chinese medicine (TCM) herb formulas, typically composed of two or more herbs, are recognized for their multicomponent, multitarget, and low-toxicity properties [9,10]. These formulas demonstrate synergistic therapeutic effects due to the interactions between multiple active ingredients, reflecting the holistic concept of TCM [11]. The combination of *Tetradium ruticarpum* (A.Juss.) T.G.Hartley as well as *Coptis chinensis* Franch is a classic traditional Chinese medicine formula, originally used to treat liver and gastrointestinal disorders [12,13,14]. *Coptis chinensis* improves obesity by inhibiting lipid accumulation [15], while *Tetradium ruticarpum* is used to treat conditions, such as vomiting and diarrhea [16]. The combined use of *Coptis chinensis* and *Tetradium ruticarpum* has been shown to effectively improve hyperlipidemia [17], cholestasis [18], and non-alcoholic fatty liver disease (NAFLD) [19]. Additionally, as bioactive components of *Coptis chinensis* and *Tetradium ruticarpum* officinalis, berberine alone can induce inguinal fat browning through the activation of AMPK and PGC-1α [20], as well as by increasing the maximum oral temperature and stimulating BAT mass [21]. Evodiamine, on the other hand, can inhibit lipid synthesis and the differentiation of C3H10T1/2 cells into adipocytes via the EGFR-PKCα-ERK signaling pathway [22,23], thereby contributing to obesity management. The combined use of *Coptis chinensis* and *Tetradium ruticarpum* has shown potential therapeutic effects in hyperlipidemia [24], NAFLD [25], depression [26], Alzheimer’s disease [25], and cancer [27]. However, it remains unclear whether the combined use of berberine and evodiamine has a synergistic effect on obesity and whether this effect is closely associated with the promotion of browning.

Therefore, this study seeks to assess the synergistic impacts of berberine and evodiamine on obesity using a high-fat diet-induced obesity model in vivo and an in vitro 3T3-L1 cell model, and to explore whether their potential mechanisms include the promotion of browning.

## 2. Results

### 2.1. The Major Components from Coptis Chinensis and Tetradium ruticarpum Inhibit Lipid Accumulation

**Figure 1:**
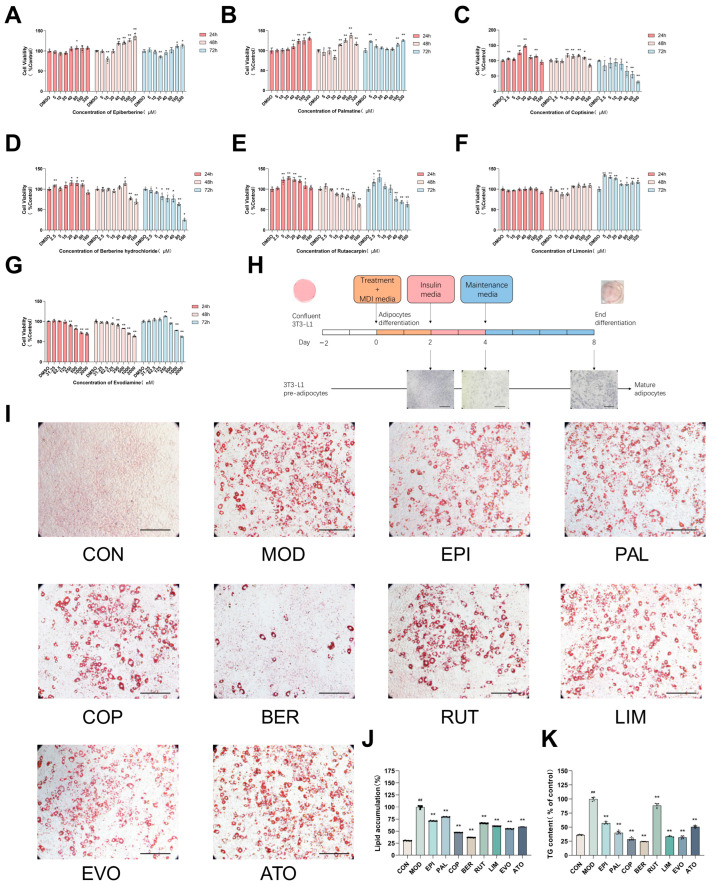
The major components from Coptis chinensis and *Tetradium ruticarpum* inhibit lipid accumulation in vitro. The cell viability was determined by CCK-8 assay in a complete medium with or without treatment of (**A**) epiberberine, (**B**) palmatine, (**C**) coptisine, (**D**) berberine hydrochloride, (**E**) rutaecarpin, (**F**) limonin, and (**G**) evodiamine (*n* = 6). (**H**) Schematic diagram of the in vitro 3T3-L1 cell assay (magnification: 20×, scale bar: 200 μm). (**I**) 3T3-L1 cell culture samples were fixed and subsequently stained with Oil Red O (*n* = 6); 20× magnification, scale bar: 200 μm. (**J**) Isopropanol quantified intracellular lipid accumulation (*n* = 6). (**K**) Triglyceride (TG) levels were measured in mature 3T3-L1 cells (*n* = 6). All data are presented as mean ± SEM, ^##^ *p* < 0.01 vs. CON; * *p* < 0.05, ** *p* < 0.01 vs. MOD.

Coptis chinensis and *Tetradium ruticarpum* contain several bioactive compounds. Seven major natural products from these two herbs were selected for preliminary experiments to identify the most effective lipid-lowering compounds. To determine the non-cytotoxic concentrations of berberine, epiberberine, coptisine, palmatine, evodiamine, rutaecarpine, and limonin, their effects on cell viability were assessed using the CCK-8 assay in 3T3-L1 cells. As shown in Figure 1A–G, berberine at concentrations ≤ 10 μM had no significant effect on 3T3-L1 cells at 24 and 48 h compared to the CON group. Similarly, epiberberine ≤ 5 μM, coptisine ≤ 5 μM, and palmatine ≤ 5 μM exhibited low cytotoxicity after 24, 48, and 72 h of treatment. Furthermore, evodiamine ≤ 0.0625 μM, rutaecarpine ≤ 5 μM, and limonin ≤ 5 μM did not affect cell viability at 24, 48, and 72 h. In light of the results, the concentrations selected for subsequent experiments were as follows: berberine (10 μM), epiberberine (5 μM), coptisine (5 μM), palmatine (5 μM), evodiamine (0.0625 μM), rutaecarpine (5 μM), and limonin (5 μM).

To further investigate the effects of these natural products on lipogenesis, a cocktail induction method was used to establish a 3T3-L1 cells model, and the cells were exposed to the designated concentrations of each compound (Figure 1H). The model group (MOD) exhibited typical enlarged, rounded cells with large lipid droplets, whereas the uninduced group (CON) displayed a fibroblast-like morphology with flat, spindle-shaped cells and no significant lipid accumulation. In contrast, treatment with the major components of Coptis chinensis and *Tetradium ruticarpum* resulted in varying degrees of differentiation abnormalities and lipid accumulation (Figure 1I). Oil Red O (ORO) staining quantified by OD values, revealed that BBE exhibited the most pronounced inhibitory effects on lipid droplets (Figure 1J). Biochemical TG assays further confirmed the significant lipid-lowering effects of BBE (Figure 1K). Therefore, berberine and evodiamine were selected as the optimal components from Coptis chinensis and Tetradium ruticarpum, respectively, for subsequent experiments.

### 2.2. Molecular Docking Analysis of BBE and Key Proteins Involved in FGF21/PGC-1α Pathway

**Figure 2:**
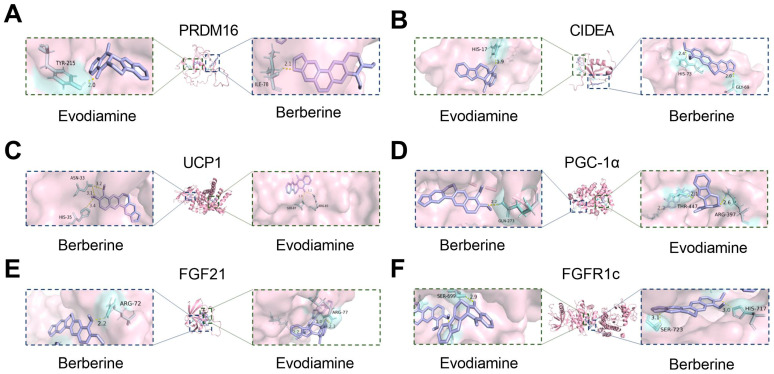
Molecular docking analysis of BBE and key proteins involved in the FGF21/PGC-1α pathway. (**A**) Molecular docking analysis of BBE with PRDM16. (**B**) Molecular docking analysis of BBE with CIDEA. (**C**) Molecular docking analysis of BBE with UCP1. (**D**) Molecular docking analysis of BBE with PGC-1α. (**E**) Molecular docking analysis of BBE with FGF21. (**F**) Molecular docking analysis of BBE with FGFR1c.

To further investigate the molecular interactions, molecular docking of BBE with PRDM16, CIDEA, UCP1, PGC-1α, FGF21, and FGFR1c was performed. The docking results, as illustrated in Figure 2A,B, revealed that the binding energy between BBE and CIDEA was −26.4 kJ/mol, and between BBE and PRDM16, was −31.0 kJ/mol. These values indicate stable binding interactions, suggesting a strong affinity between BBE and both PRDM16 and CIDEA. The negative binding energies indicate spontaneous binding and stable conformations, which support the observed biological effects of BBE on browning. Furthermore, the docking binding energies for BBE with other key proteins involved in the FGF21/PGC-1α pathway were as follows: UCP1 with BBE, −31.4 kJ/mol; PGC−1α with BBE, −30.5 kJ/mol; FGF21 with BBE, −26.4 kJ/mol; and FGFR1c with BBE, −28.9 kJ/mol (Figure 2C–F). These binding energies also indicate spontaneous binding and stable conformations, further supporting the biological effects of BBE on thermogenesis and the activation of the FGF21/PGC-1α pathway.

### 2.3. BBE Promotes Browning and Improves Intracellular Lipid Profiles in 3T3-L1 Cells

**Figure 3:**
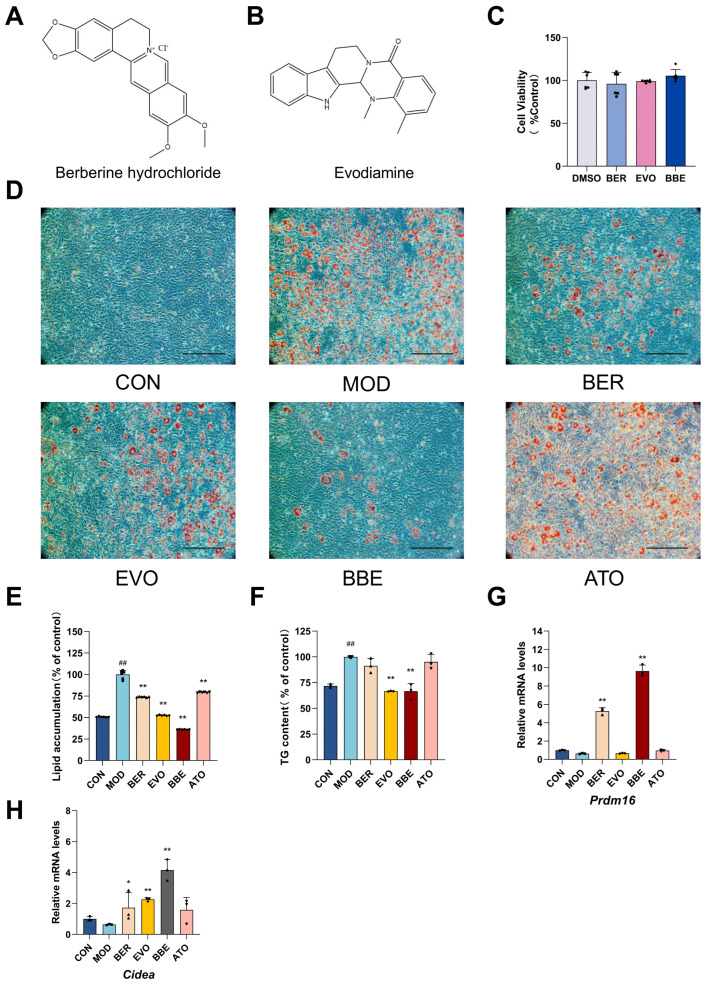
BBE promotes browning and ameliorates lipid accumulation. (**A**) Chemical structure of berberine hydrochloride. (**B**) Chemical structure of evodiamine. (**C**) Cell viability was assessed using the CCK8 in complete medium (*n* = 6). (**D**) 3T3-L1 cells were subjected to staining with Oil Red O with or without treatment of BER, EVO, or BBE with or without treatment of BER, EVO, or BBE (*n* = 6); 20× magnification, scale bar: 200 μm. (**E**) Oil Red O staining was performed on mature 3T3-L1 adipocytes (*n* = 6). (**F**) Lipid deposition was quantified by dissolving Oil Red O-stained lipid droplets in isopropanol in 3T3-L1 cells (*n* = 3). (**G**) qRT-PCR was applied to analyze *PRDM16* gene expression (*n* = 3). (**H**) qRT-PCR was employed to evaluate *CIDEA* gene expression (*n* = 3). Values are shown as mean ± SEM. ^##^ *p* < 0.01 vs. CON; * *p* < 0.05, ** *p* < 0.01 vs. MOD.

Adipogenesis primarily involves the differentiation of adipocytes and the accumulation of triglycerides, leading to fat tissue expansion and energy storage. To evaluate the synergistic effects of BBE on inhibiting lipogenesis and promoting adipocyte browning, 3T3-L1 cells were used as an in vitro model, with comparisons made to single treatments. First, non-cytotoxic concentrations of 10 μM berberine (Figure 3A) and 62.5 nM evodiamine (Figure 3B) were confirmed to be safe when co-applied (Figure 3C). To further explore the impact of BBE on lipid metabolism, the 3T3-L1 cells model was used to assess its effects on intracellular lipid accumulation and browning. Microscopic observations revealed that BBE significantly inhibited lipid accumulation during 3T3-L1 differentiation more effectively than berberine or evodiamine alone (Figure 3D,E). Quantification of intracellular triglyceride (TG) content further confirmed that BBE significantly suppressed lipid accumulation compared to the single treatments (Figure 3F). Moreover, BBE treatment significantly upregulated the expression of browning-related markers, *Prdm16* and *Cidea*, at the mRNA levels (Figure 3G,H). Data from this study suggest that BBE exhibits a more robust ability to inhibit lipid droplet formation and to promote adipocyte browning compared to individual treatments.

### 2.4. BBE Enhances Thermogenesis in Differentiated 3T3-L1 Cells

**Figure 4:**
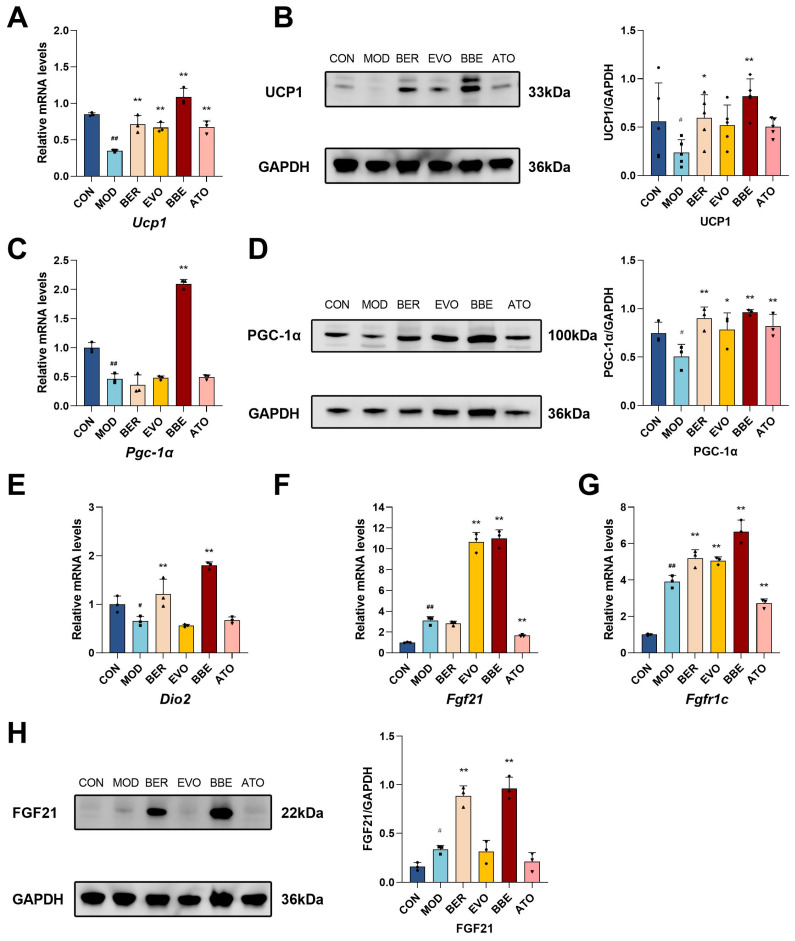
BBE enhances thermogenesis in vitro. (**A**) qRT-PCR was utilized to examine *UCP1* gene expression levels (*n* = 3). (**B**) The expression of UCP1 protein was examined using Western blot techniques (*n* = 5). (**C**) The levels of *PGC-1α* gene expression were quantified using qRT-PCR (*n* = 3). (**D**) PGC-1α protein expression was evaluated using Western blot (*n* = 3). (**E**) *DIO2* gene expression was assessed using qRT-PCR (*n* = 3). (**F**) qRT-PCR was conducted to analyze *FGF21* gene expression (*n* = 3). (**G**) qRT-PCR analysis of *FGFR1c* gene expression (*n* = 3). (**H**) The expression of FGF21 protein was assessed by Western blot (*n* = 3). The values are shown as mean ± SEM. ^#^ *p* < 0.05, ^##^ *p* < 0.01 vs. CON; * *p* < 0.05, ** *p* < 0.01 vs. MOD.

Adipocyte thermogenesis is a crucial and relatively independent physiological process that converts stored chemical energy into heat through non-shivering mechanisms, playing an essential role in energy expenditure and offering potential benefits for obesity management. To explore the effects of BBE on thermogenesis, we analyzed the expression of thermogenesis-related genes and browning-associated secretory factors in 3T3-L1 cells. Quantitative reverse transcription polymerase chain reaction (qRT-PCR) analysis demonstrated that BBE treatment substantially increased the mRNA expression profiles of *Ucp1*, *Pgc-1α*, and *Dio2* relative to monotherapy with berberine or evodiamine (Figure 4A,C,E). Western blot analysis further confirmed that BBE markedly enhanced the protein expression of PGC-1α and UCP1 in 3T3-L1 cells (Figure 4B,D).

Additionally, BBE treatment significantly increased the mRNA expression of *Fgf21* and *Fgfr1c* (Figure 4F,G) and upregulated FGF21 protein expression (Figure 4H). These results demonstrate that BBE promotes adipocyte thermogenesis and browning by activating key thermogenic genes and secretory factors.

### 2.5. BBE Reduces Body Weight and Enhances Metabolic Health in Mice Maintained on a High-Fat Diet Regimen

**Figure 5:**
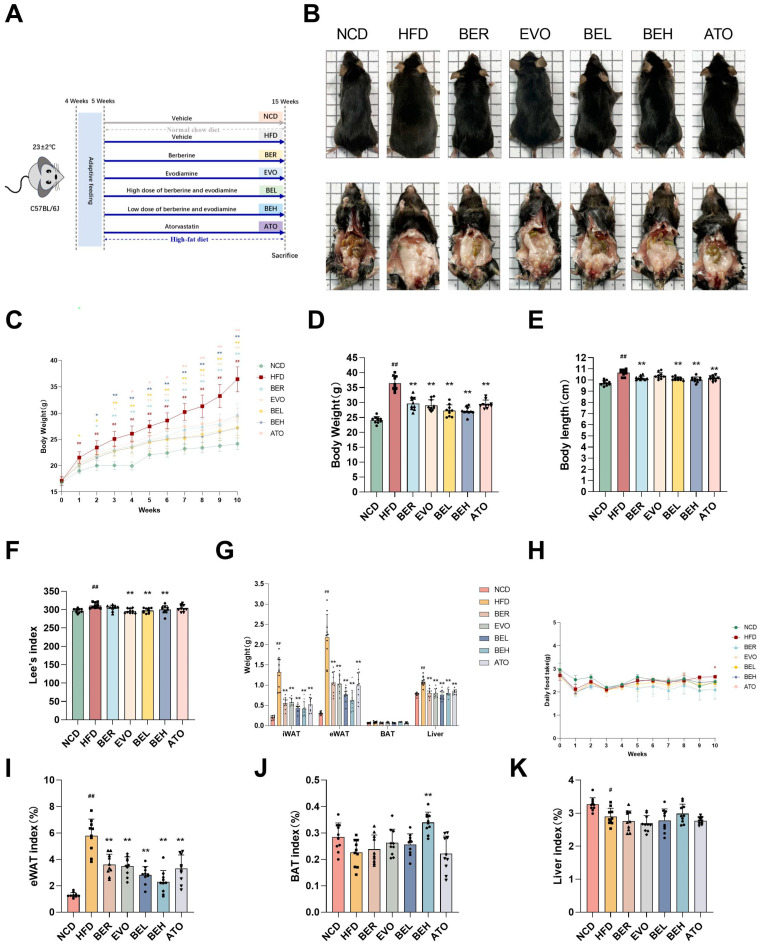
BBE reduces body weight and fat mass in vivo. (**A**) Experimental design for dietary and BBE treatment in HFD-induced obese mice. (**B**) Representative gross morphological images of the dorsal and ventral views of the mice before sacrifice (*n* = 10). (**C**) Body weight changes during the experiment (*n* = 10). (**D**) Comparison of body weights at the end of 10 weeks (*n* = 10). (**E**) Comparison of body lengths at the end of 10 weeks (*n* = 10). (**F**) Lee’s index comparison among NCD, HFD, BER, EVO, BBE, and ATO groups (*n* = 10). (**G**) Alterations in the mass of iWAT, eWAT, BAT, and liver were observed (*n* = 10). (**H**) Food intake of mice from weeks 0 to 10 (*n* = 10). (**I**) eWAT index of different groups (*n* = 10). (**J**) BAT index of different groups (*n* = 10). (**K**) Liver index of different groups (*n* = 10). Data are expressed as mean ± SEM. ^#^ *p* < 0.05, ^##^ *p* < 0.01 vs. NCD; * *p* < 0.05, ** *p* < 0.01 vs. HFD.

To investigate the anti-obesity effects of BBE, a control group of mice fed a normal-fat diet (NCD) was established alongside an experimental group whose diet consisted of high-fat content (HFD) over a period of ten weeks to develop an obese model using C57BL/6J mice. Berberine (33 mg/kg), evodiamine (8 mg/kg), a low-dose combination of berberine and evodiamine (BEL, 33 mg/kg + 8 mg/kg), and a high-dose combination (BEH, 132 mg/kg + 32 mg/kg) were administered. Atorvastatin (1.5 mg/kg) was used as a positive control (Figure 5A). After 10 weeks of treatment, both the BEL and BEH groups exhibited significantly smaller body sizes and reduced fat pad sizes compared to the HFD group (Figure 5B). From the second week onward, BBE-treated mice exhibited significantly reduced weight gain compared to the HFD group (Figure 5C). By the end of the experiment, the HFD group showed a body weight increase of over 20% compared to the NCD group, confirming the successful establishment of the obesity model. In contrast, both the BEL and BEH groups showed significantly lower body weights than the HFD group (Figure 5D). Additionally, body length and Lee’s index were significantly reduced in the BEL and BEH groups compared to the HFD group (Figure 5E,F). Food intake remained similar across all groups, with only a slight difference observed between the NCD and HFD groups (Figure 5H).

Notably, BBE treatment significantly reduced the weights of epididymal white adipose tissue (eWAT), inguinal white adipose tissue (iWAT), and liver tissues that were increased by the high-fat diet (Figure 5G). The eWAT index showed a marked increase in the HFD group, whereas the BAT index and liver index demonstrated a notable decrease. In contrast, the BEL and BEH groups showed significantly reduced eWAT indices and increased BAT and liver indices (Figure 5I–K). Despite the absence of a notable disparity in brown adipose tissue weight, the brown fat index underwent a substantial augmentation with BBE treatment (Figure 5J). These results indicate that BBE effectively reduces fat mass and improves metabolic health in HFD-induced obese mice.

**Figure 6:**
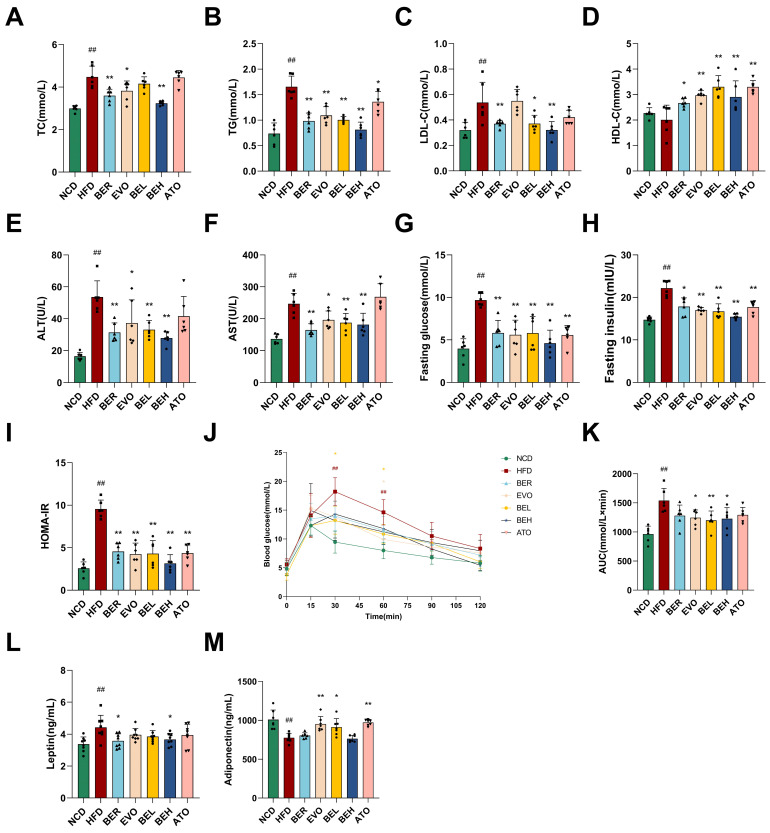
BBE improves lipid and glucose metabolism while ameliorating insulin resistance. The serum lipid profiles of (**A**) TC, (**B**) TG, (**C**) LDL-C, (**D**) HDL-C, (**E**) ALT, and (**F**) AST (*n* = 6). (**G**) Fasting blood glucose concentrations (*n* = 6). (**H**) Fasting insulin levels (*n* = 6). (**I**) Homeostatic model assessment of insulin resistance (HOMA-IR) values (*n* = 6). (**J**,**K**) Glucose tolerance tests were conducted in mice, with the area under the curve (AUC) analyzed (*n* = 6). (**L**) Serum leptin levels were estimated using ELISA kits (*n* = 6). (**M**) Serum adiponectin levels were estimated using ELISA kits (*n* = 6). Data are expressed as mean ± SEM. ^##^ *p* < 0.01 vs. NCD; * *p* < 0.05, ** *p* < 0.01 vs. HFD.

Moreover, the impact of BBE treatment on lipid and glucose metabolism, as well as insulin resistance, was assessed. After 10 weeks of HFD feeding, the BEL and BEH groups exhibited significantly lower serum levels of TC, LDL-C, and TG compared to the HFD group (Figure 6A–C). In contrast, HDL-C levels were markedly elevated (Figure 6D). Serum ALT and AST concentrations, which serve as markers of liver function, were substantially decreased in the BBE-treated groups, indicating enhanced hepatic health (Figure 6E,F). Fasting blood glucose and insulin concentrations were markedly increased in the HFD group, whereas BBE treatment significantly enhanced insulin levels (Figure 6G,H). The HOMA-IR, an index of insulin resistance, demonstrated that BBE treatment markedly improved insulin sensitivity (Figure 6I). Glucose tolerance tests revealed that BBE reversed the high blood glucose levels and the AUC induced by the HFD, indicating improved glucose tolerance (Figure 6J,K). Additionally, adipokine levels were measured, and BBE treatment significantly increased adiponectin levels while decreasing leptin levels (Figure 6L,M). These findings suggest that BBE can improve insulin sensitivity and overall metabolic status by regulating adipokine secretion, thereby reversing HFD-induced dyslipidemia and glucose metabolism disorders.

In summary, our results demonstrate that the combination of berberine and evodiamine effectively reduces body weight and fat mass, improves lipid and glucose metabolism, and enhances insulin sensitivity in vivo, highlighting the potential of BBE as a therapeutic agent for obesity and related metabolic disorders.

### 2.6. BBE Reduces Adipocyte Size in Subcutaneous Fat and Lipid Accumulation in Liver

**Figure 7:**
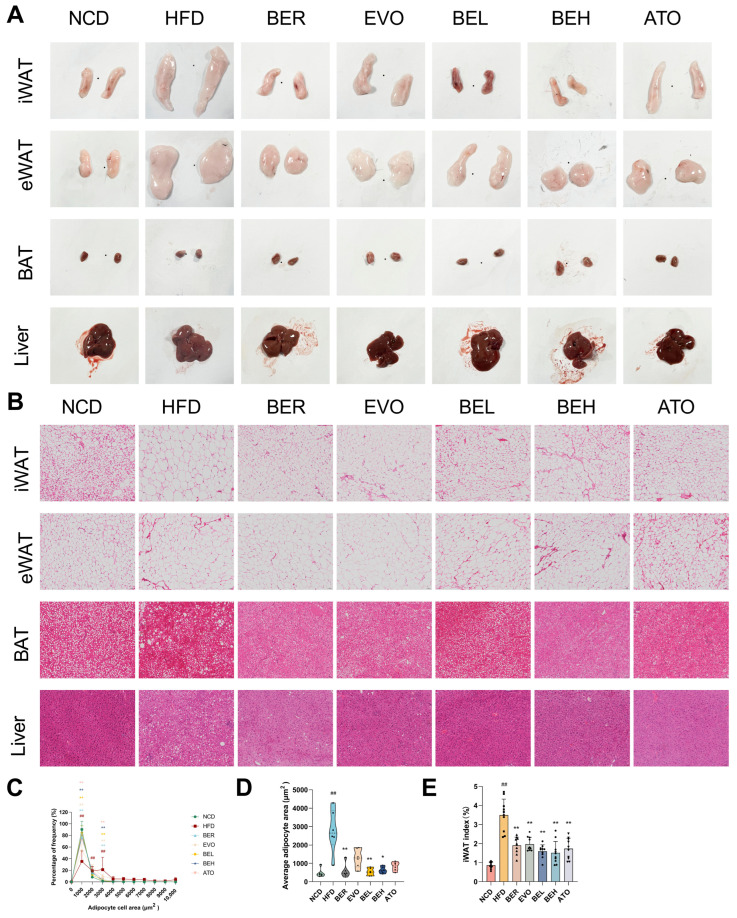
BBE reduces adipocyte size in subcutaneous fat and attenuates lipid accumulation in liver tissue in vivo. BBE reduces adipocyte size in subcutaneous fat and lipid deposition in liver tissue of HFD-induced mice. (**A**) Representative photographs of iWAT, BAT, eWAT, and liver (*n* = 6). (**B**) H&E staining of eWAT, iWAT, BAT, and liver (*n* = 6); 20× magnification, scale bar: 50 μm. (**C**,**D**) Adipocyte size profiling of iWAT (*n* = 6). (**E**) iWAT index among NCD, HFD, BER, EVO, BBE, and ATO groups (*n* = 6). The data are expressed as mean ± SEM. ^##^ *p* < 0.01 vs. NCD; * *p* < 0.05, ** *p* < 0.01 vs. HFD.

To assess the histological effects of BBE on fat and liver tissues, Hematoxylin and eosin (H&E) staining was performed on iWAT, eWAT, and BAT from HFD-fed mice. Compared to the lean NCD group, the HFD group exhibited enlarged iWAT, eWAT, and depot sizes, dominated by hypertrophic adipocytes and hyperplastic tissue (Figure 7A). However, BBE treatment significantly reduced the adipocyte diameter in iWAT, eWAT, and BAT, with the most pronounced changes observed in iWAT (Figure 7B). The iWAT area was significantly reduced, accompanied by a significant increase in smaller-diameter adipocytes, leading to a lower iWAT index (Figure 7C–E). In the liver, BBE treatment significantly reduced lipid accumulation and alleviated hepatic steatosis (Figure 7B). These results indicate that BBE effectively improves the quality of white adipose tissue (WAT) and hepatic steatosis in HFD-fed mice.

### 2.7. BBE Promotes iWAT Browning in Obese Mice

**Figure 8:**
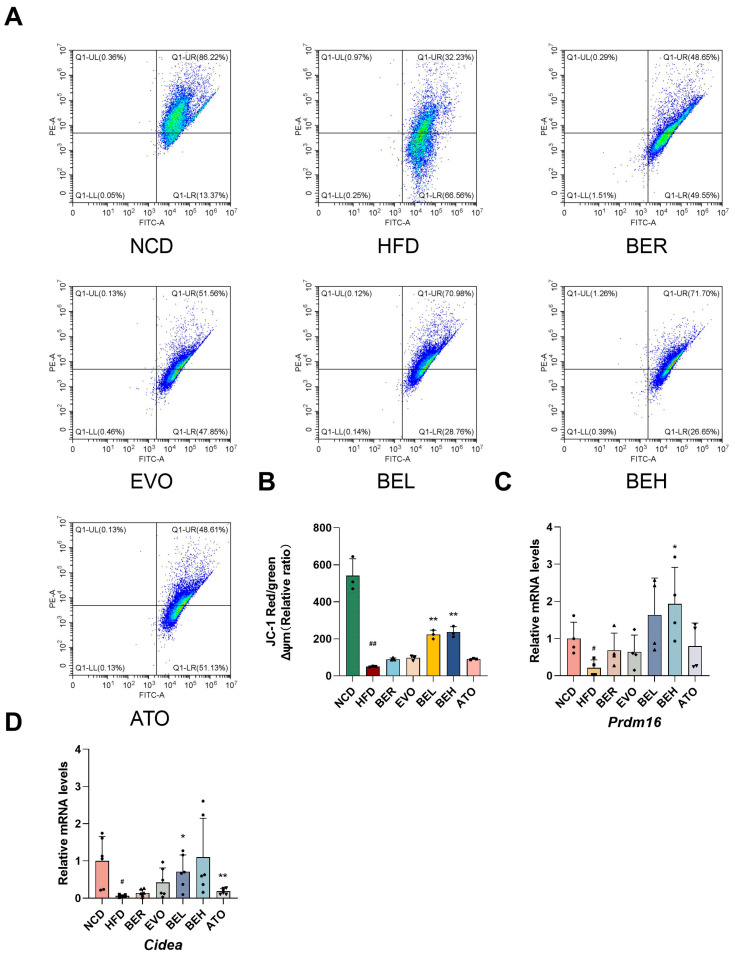
BBE promotes iWAT browning in obese mice. (**A**) Flow cytometric analysis of mitochondrial depolarization in inguinal adipose tissue (*n* = 3). (**B**) Quantification of mitochondrial membrane potential (ΔΨm) by flow cytometry (*n* = 6). (**C**) The expression levels of the *PRDM16* gene were measured within inguinal adipose tissue samples employing qRT-PCR. (*n* = 4). (**D**) The expression of the *CIDEA* gene was assessed in inguinal adipose tissue via qRT-PCR. (*n* = 6). Values are expressed as mean ± SEM. ^#^ *p* < 0.05, ^##^ *p* < 0.01 vs. NCD; * *p* < 0.05, ** *p* < 0.01 vs. HFD.

This study further explored the impact of BBE on the browning of white adipose tissue. Browning converts white adipocytes into beige cells that function similarly to brown adipocytes, involving changes in mitochondrial membrane potential. To assess this, JC-1 fluorescence analysis was performed. JC-1, a sensitive fluorescent probe, has been utilized to monitor mitochondrial membrane potential. Aggregates formed by JC-1 emit red fluorescence in polarized mitochondria, whereas depolarized mitochondria exhibit green fluorescence. The results revealed a marked increase in red fluorescence and a corresponding decrease in green fluorescence within white adipose tissue, unequivocally demonstrating that BBE treatment significantly augmented mitochondrial membrane potential, suggesting increased mitochondrial activity and the browning of adipocytes (Figure 8A,B). Simultaneously, BBE treatment significantly upregulated the expression of *Cidea* and *Prdm16* genes, which are key regulatory markers of adipocyte browning (Figure 8C,D). Collectively, these findings suggest that BBE not only promotes adipocyte browning but may also enhance mitochondrial function by improving mitochondrial membrane potential, providing a potential basis for obesity treatment.

### 2.8. BBE Promotes iWAT Thermogenesis In Vivo

**Figure 9:**
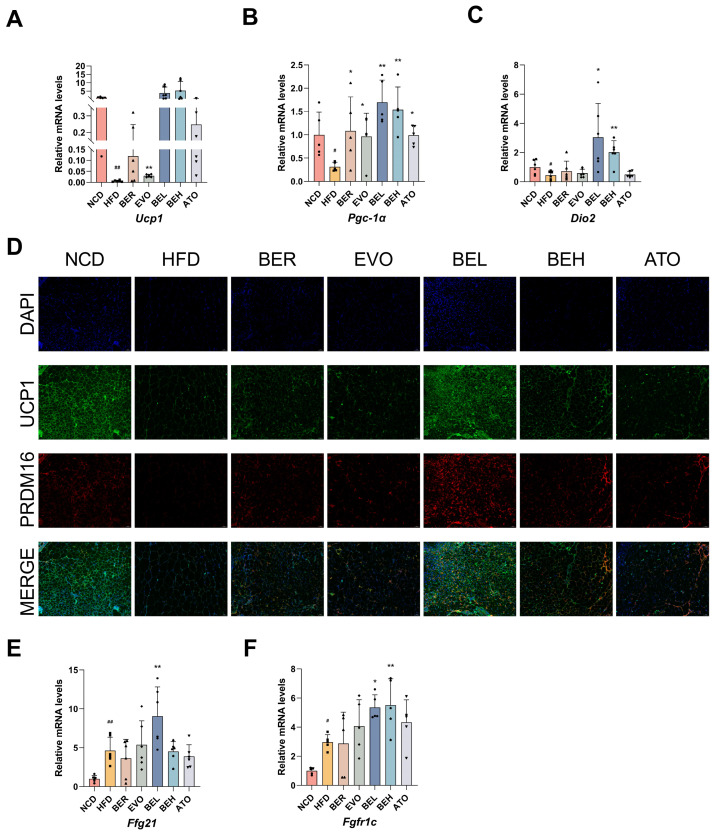
BBE promotes iWAT thermogenesis in obese mice. (**A**) qRT-PCR assessment of *UCP1* mRNA levels in inguinal adipose tissue (*n* = 6). (**B**) qRT-PCR analysis of *PGC-1α* mRNA expression in iWAT (*n* = 5). (**C**) qRT-PCR analysis of *DIO2* mRNA expression in iWAT (*n* = 6). (**D**) Immunofluorescence microscopy images illustrating UCP1+ and PRDM16+ cells in iWAT (*n* = 3); 20× magnification, scale bar: 50 μm. Green: UCP1-positive staining. Red: PRDM16-positive staining. (**E**) qRT-PCR measurement of *FGF21* gene expression in iWAT (*n* = 6). (**F**) qRT-PCR investigation of *FGFR1c* mRNA expression in inguinal adipose tissue (*n* = 5). The values are represented as mean ± SEM. ^#^ *p* < 0.05, ^##^ *p* < 0.01 vs. NCD; * *p* < 0.05, ** *p* < 0.01 vs. HFD.

To further examine the effects of BBE on adipose tissue browning and thermogenesis in obese mice, we compared the BBE-treated group to the HFD group. The expression of thermogenic genes *Ucp1*, *Pgc-1α*, and *Dio2* was significantly upregulated in the BBE intervention group, as shown in Figure 9A–C. Furthermore, immunofluorescence staining was performed to examine UCP1 and PRDM16 proteins in iWAT. The results revealed that the green fluorescence intensity of the UCP1 protein and the red fluorescence intensity of the PRDM16 protein were significantly elevated in both the low- and high-dose BBE groups, with a notable increase in their co-localization. Following BBE treatment, the increased expression of UCP1-positive beige adipocytes suggests the activation of browning processes in iWAT depot. These findings indicate that BBE can augment the expression of genes and proteins associated with thermogenesis in mouse iWAT, as illustrated in Figure 9D. Additionally, it has been reported that FGF21 is closely associated with improvements in circulating cholesterol, free fatty acids, blood glucose levels, and increased insulin sensitivity [28]. Our experimental results demonstrated that the expression of *Fgf21* and *Fgfr1c* genes was significantly elevated following BBE administration, as shown in Figure 9E,F. In brief, these findings reveal that BBE may occupy a significant position in ameliorating obesity by promoting browning and enhancing thermogenesis.

## 3. Discussion

Obesity is a prolonged imbalance between energy intake and expenditure, and has become a global epidemic [29], currently standing as the fourth most significant risk factor for global mortality [30]. Beyond its immediate health implications, obesity significantly contributes to the development of type 2 diabetes, liver cancer [31], and multiple myeloma, highlighting the urgent need to explore diverse, safe, and effective weight loss mechanisms. Recent studies have shown the adverse side effects of current clinical obesity medications, such as steatorrhea, oily spotting [32], malabsorption of fat-soluble vitamins [33], and pancreatitis [34]. Natural products, particularly those derived from traditional Chinese medicine, offer a promising alternative with high efficacy and minimal side effects [35]. Our findings demonstrate that the co-administration of berberine and evodiamine significantly improves physiological parameters, such as body weight, reduces fat accumulation, alleviates hepatic steatosis, and enhances glucose and lipid metabolism, while also regulating adipokine levels. In vitro experiments further support these results, showing that the combination of berberine and evodiamine is found to significantly inhibit lipid accumulation within 3T3-L1 cells. To elucidate the underlying mechanism, we found that BBE plays a key role in ameliorating obesity by activating the FGF21/PGC-1α pathway and promoting browning. In conclusion, our study reveals a new mechanism by which the combination of berberine and evodiamine exerts a synergistic effect to effectively improve obesity.

*Coptis chinensis* and *Tetradium ruticarpum*, two herbs with a long history in Chinese medicine, are known for their bioactive compounds that possess lipid-lowering, glucose-lowering, anti-inflammatory, and anti-viral properties [36]. The combination of these herbs, as recorded in the Taiping Shenghui Fang during the Song Dynasty, has been traditionally used in a 1:1 ratio to treat various metabolic disorders [37,38,39]. Bioactive constituents of *Coptis chinensis*, such as berberine, coptisine, epiberberine, and palmatine [40], have been reported to decrease body weight and enhance insulin sensitivity, and inhibit adipose tissue inflammation in HFD-fed mice [41]. Similarly, active components of *Tetradium ruticarpum*, including evodiamine, rutaecarpine, and limonin [42], have demonstrated potential in improving obesity, lowering blood pressure [43], and exhibiting anti-tumor effects [44]. Notably, berberine can promote the differentiation of brown preadipocytes via the AMPK-PRDM16 axis [20], inhibit TLR4/TNF-α-mediated inflammatory responses, and reduce the expression of iNOS and COX-2 [28,45,46], thereby enhancing thermogenesis and alleviating obesity through improved inflammation. Evodiamine, on the other hand, inhibits adipocyte differentiation by blocking the ERK/MAPK pathway and modulates the NF-κB signaling pathway to reduce inflammatory responses [22,23,47], demonstrating significant anti-obesity effects. Since it is unclear whether berberine and evodiamine have potential synergistic effects in improving obesity, this study aims to explore their potential molecular mechanisms using an obesity mice model and 3T3-L1 cell model.

Accumulating clinical trial data and consistent preclinical findings support the metabolic benefits of berberine and evodiamine. Clinical trials have demonstrated that berberine at doses of 1000–1200 mg/day for 3–6 months significantly improves glycemic and lipid profiles and provides renal protection in patients with type 2 diabetes mellitus (T2DM), without serious adverse effects [48,49]. In rodent models, berberine has been widely used at doses ranging from 50 to 250 mg/kg, showing consistent efficacy in improving insulin sensitivity, lipid metabolism, and glucose homeostasis [50,51,52]. Similarly, evodiamine has demonstrated therapeutic potential at doses of 10–20 mg/kg in various rodent models of obesity and metabolic dysfunction, including improvements in glucose and lipid metabolism [23,53]. Notably, a previous study reported that the combined administration of 72.6 mg/kg berberine and 16.6 mg/kg evodiamine produced synergistic lipid-lowering effects in obese rats [24], thereby supporting our combinatorial approach. Although both agents exhibit favorable safety profiles, the lowest observed adverse effect level (LOAEL) for berberine in mice is as high as 841 mg/kg/day, indicating a broad therapeutic window [54]. In our study, berberine and evodiamine were administered at 66 mg/kg and 16 mg/kg, respectively, via oral gavage, either alone or in combination. When adjusted for human equivalent dosing using standard interspecies scaling, this corresponds to approximately 0.63 g/day, which is significantly lower than the doses previously used in clinical trials. Consistent with previous reports, berberine (0–10 μM) effectively suppresses preadipocyte differentiation and adipogenesis in human omental adipose-derived stromal cells [55]. Separately, evodiamine (0.01–1 μM) demonstrates anti-adipogenic activity in both 3T3-L1 and 10T1/2 cell lines [23], and evodiamine (0–20 μM) exhibits anti-inflammatory effects in primary mouse chondrocytes [47]. Therefore, the selected dosing regimen demonstrates pharmacological efficacy, favorable safety, and clinical translatability, providing a robust foundation for future translational studies and mechanistic investigations.

The 2020 edition of the Chinese Pharmacopoeia identifies berberine and evodiamine as representative natural compounds found in Coptis chinensis and Tetradium ruticarpum, respectively. Extensive modern research has confirmed that berberine and evodiamine are the main active components of Coptis chinensis and *Tetradium ruticarpum* [56,57]. Berberine has been shown to have multiple therapeutic effects in treating dyslipidemia, hyperglycemia, and hepatic steatosis [58], while evodiamine exhibits significant benefits in improving diabetes and lipid metabolism [53,59]. In 3T3-L1 cells, we demonstrated that natural components from *Coptis chinensis* and *Tetradium ruticarpum* effectively inhibit adipogenesis, with the concurrent use of berberine and evodiamine exhibiting the strongest effect. And in obesity, adipocyte size significantly increases, accompanied by lipid droplet accumulation, marking adipocyte maturation [60]. Based on the 48 h dosing data, treatments with berberine (10 μM), epiberberine (5 μM), coptisine (5 μM), palmatine (5 μM), evodiamine (0.0625 μM), rutaecarpine (5 μM), and limonin (5 μM) did not affect cell viability, allowing these concentrations to be used in subsequent experiments. The combined administration of berberine and evodiamine demonstrated the most significant reduction in lipid droplet accumulation and triglyceride synthesis. This effect suggests that the synergistic interaction between these two compounds may enhance their anti-adipogenic properties.

In this study, we used 4-week-old C57BL/6J mice to investigate the anti-obesity effects of the combination of berberine and evodiamine in HFD-induced obesity. Initial experiments revealed a transient decrease in food intake during the first week, though body weight continued to rise. This may be due to the mice’s adaptation to the high-fat diet and normal diet, where even with reduced food intake, their energy needs for growth and development were still being met. Furthermore, obesity is not only characterized by abnormal weight gain but is more critically defined by excessive adipose tissue accumulation, dyslipidemia, impaired glucose tolerance, and elevated fasting insulin levels [61]. Our findings demonstrated that the combination of berberine and evodiamine treatment significantly reduced adipose mass and improved serum lipid profiles, fasting insulin levels, and glucose tolerance in HFD-fed mice. These results underscore the therapeutic potential of the combination of berberine and evodiamine in targeting not just weight gain but also the broader metabolic disorders associated with obesity. Notably, previous studies have shown that adipocyte-specific deletion of CAMK2 improves insulin signaling and glucose homeostasis in obese models, further highlighting the critical role of adipose tissue in systemic metabolic regulation [62]. In our study, molecular docking revealed high-affinity binding sites between BBE components and the PRDM16 and CIDEA proteins. The browning of white adipose tissue involves the differentiation of beige adipocytes, mitochondrial biogenesis, and non-shivering thermogenesis, regulated by transcription factors, such as PRDM16, PGC-1α, and UCP1 [63,64]. H&E staining of inguinal and epididymal adipose tissues showed that BBE treatment significantly reduced adipocyte size and increased the number of small adipocytes, likely representing newly differentiated cells, with more pronounced changes in inguinal fat. Given the critical role of ΔΨm in assessing mitochondrial viability and function, the restoration of ΔΨm is essential for improving metabolic health [65,66,67]. The significant reversal of the mitochondrial membrane potential in BBE-treated inguinal white adipocytes suggests an improved mitochondrial function and activity, a key feature in the browning process [68]. Berberine and evodiamine significantly upregulate the expression of PRDM16 and CIDEA, key transcriptional regulators of beige fat development in WAT [69,70]. PRDM16 is a key transcriptional switch in the phenotypic transformation of brown fat, and the upregulation of the above key transcriptional regulators confirms the role of BBE in promoting the browning of WAT.

The FGF21/PGC-1α signaling axis is a critical component of thermogenic signaling cascades. FGF21 expression is elevated in both obese humans and mice [71], and the exogenous administration of FGF21 has been shown to mitigate obesity by reducing inflammation, promoting the browning of white adipose tissue, and enhancing heat production [72,73]. Mechanistically, FGF21 binds to receptor complexes, such as FGFR1c, thereby activating the p38 MAPK signaling pathway, which subsequently enhances PGC-1α activity and induces UCP1 expression [74,75,76,77,78]. PGC-1α, a hallmark of beige adipocyte differentiation, is critical for mitochondrial biogenesis and thermogenic programming [79]. It promotes mitochondrial DNA replication and transcription [80] and interacts with nuclear receptors, such as PPARγ, to drive UCP1 expression, thereby boosting thermogenesis and energy expenditure [81]. In our study, administration of the combination of berberine and evodiamine reversed the downregulation of key brown fat markers (*Prdm16*, *Pgc-1α*) and thermogenic genes (*Ucp1*, *Cidea*, *Dio2*) in differentiated 3T3-L1 adipocytes. BBE also significantly upregulated the gene expression of *Fgf21* and *Fgfr1c*. At the protein level, the combination of berberine and evodiamine treatment enhanced the expression of FGF21, PGC-1α, and UCP1 in 3T3-L1 cells, and immunofluorescence analysis confirmed increased levels of UCP1 and PRDM16 in iWAT of obese mice. These findings substantiate that the combination of berberine and evodiamine exerts its anti-obesity effects, which are mechanistically linked to the activation of the FGF21/PGC-1α signaling pathway and the subsequent promotion of adipose tissue browning.

However, adipose browning is a multifaceted process involving numerous molecular regulators beyond FGF21/PGC-1α. For example, AMP-activated protein kinase (AMPK) activation enhances mitochondrial biogenesis and thermogenesis through the AMPK–SIRT1–PGC-1α signaling cascade, leading to increased UCP1 expression [82,83]. β3-adrenergic receptor (β3-AR) stimulation also induces the cAMP–PKA pathway, which recruits beige adipocytes and promotes thermogenic gene expression in WAT [84,85,86]. In addition, the activation of farnesoid X receptor (FXR) and Takeda G-protein-coupled receptor 5 (TGR5) by bile acids has been shown to increase GLP-1 secretion and hepatic FGF21 production, thereby enhancing systemic energy expenditure and adipose browning [87,88]. Although our findings demonstrate that FGF21/PGC-1α is the primary mechanistic target of BBE, the involvement of additional regulatory pathways cannot be excluded. Specifically, we did not directly assess cellular respiration or energy metabolism, such as oxygen consumption rate (OCR) in adipocytes or whole-body energy expenditure in mice. These limitations hinder our ability to definitively link BBE-induced browning to enhanced thermogenesis or caloric expenditure. Nonetheless, the observed reductions in body weight, inguinal and epididymal fat mass, hepatic steatosis, and the upregulation of browning-related genes and mitochondrial activity collectively indicate that the combination of berberine and evodiamine ameliorates obesity by promoting adipose browning and mitochondrial function.

In summary, the combination of berberine and evodiamine represents a promising therapeutic strategy for the management of obesity. BBE activates the FGF21/PGC-1α axis, enhances white adipose browning, and stimulates thermogenesis, contributing to improved metabolic outcomes. While these findings are encouraging, further studies are needed to confirm the contribution of other thermogenic signaling cascades, such as AMPK, β3-AR, and bile acid-FXR/TGR5 pathways. Future research will include direct measurements of energy expenditure, such as oxygen consumption and CO_2_ production using metabolic cages, and OCR in isolated adipose-derived stem cells. Additionally, targeted inhibition of specific signaling nodes and the application of surface plasmon resonance (SPR) analysis will be employed to dissect the molecular interactions between BBE components and their protein targets. These systematic investigations will elucidate the mechanistic basis of BBE’s anti-obesity effects and evaluate its potential therapeutic applications.

## 4. Materials and Methods

### 4.1. Drugs and Chemical Reagents

Berberine hydrochloride (BER, HPLC ≥ 98%, A0151), Palmatine hydrochloride (PAL, HPLC ≥ 98%, A0031), and Epiberberine (EPI, HPLC ≥ 98%, A0627) were purchased from MUST (CHENG DU) BIOTECHNOLOGY Co., Ltd. (Chengdu, China). Rutaecarpine (RUT, HPLC ≥ 98%, PS0077) and Coptisine chloride (COP, HPLC ≥ 98%, PS1962) were obtained from Push Bio-technology Co., Ltd. (Chengdu, China). Evodiamine (EVO, HPLC ≥ 98%, S31472) and Limonin (LIM, HPLC ≥ 98%, S31594) were procured from Shanghai Yuanye Bio-Technology Co., Ltd. (Shanghai, China). Atorvastatin Calcium Tablets were sourced from Beijing Jialin Pharmaceutical Co., Ltd. (Beijing, China). Isoflurane (R510-22-10) was obtained from Reward Life Technology Co., Ltd. (Shenzhen, China). Biochemical kits for triglyceride (TG, 0041-30-52925), total cholesterol (TC, 0041-30-52924), aspartate aminotransferase (AST, 105-000443-00), low-density lipoprotein cholesterol (LDL-C, 105-000464-00), glucose (GLU, 0041-30-42193), alanine aminotransferase (ALT, 105-000442-00), and high-density lipoprotein cholesterol (HDL-C, 105-000463-00) were procured from Mindray Bio-medical Electronics Co. (Shenzhen, China). Dulbecco’s modified Eagle’s medium (DMEM) was sourced from Gibco (Grand Island, NY, USA). Fetal bovine serum (FBS, 04-001-1ACS) was obtained from Biological Industries (Beit Haemek, Israel). 3-Isobutyl-1-methylxanthine was obtained from Sigma-Aldrich (28822-58-4, St. Louis, MO, USA). Insulin (P3376) was purchased from Beyotime Biotechnology Co., Ltd. (Shanghai, China). CCK-8 cell proliferation and cytotoxicity assay kit (CA1210), penicillin-streptomycin liquid (P1400), dexamethasone (D8040), and Oil Red O stain kit (G1262) were obtained from Beijing Solarbio Science & Technology Co., Ltd. (Beijing, China). PBS (G4207), RIPA lysis buffer (G2002), and DAPI stain solution were procured from Servicebio Technology Co., Ltd. (Wuhan, China). BCA protein assay kit (AR1189) was obtained from Boster Biological Technology Co., Ltd. (Pleasanton, CA, USA). UCP1 antibody (23673-1-AP) was procured from Proteintech Biotechnology Co., Ltd. (Wuhan, China). PGC-1α antibody (A20995), FGF21 antibody (A21463), HRP-conjugated goat anti-rabbit IgG (H+L) antibody (AS014), and GAPDH antibody (A19056) was bought by ABclonal Biotechnology Co., Ltd., located in Wuhan, China. The PRDM16 antibody (bs-19986R) was sourced by BIOSS Biotechnology Co., Ltd., Beijing, China. Multicolor prestained protein ladder (WJ103), Omni-ECL Femto Light Chemiluminescence Kit (SQ201), and Omni-Easy Protein Sample Loading Buffer (LT101S) were sourced from Epizyme Biotech Co., Ltd. (Shanghai, China).

### 4.2. Cell Culture and Cell Viability

3T3-L1 cells were kindly provided by Cell Bank, Chinese Academy of Sciences, and cultured in a humidified incubator at 37 °C with 5% CO_2_. The cells were maintained in complete growth medium consisting of 1% penicillin-streptomycin (P/S), 89% DMEM, and 10% FBS. Cells were inoculated into 96-well plates at a concentration of 3 × 10^4^ cells/mL. Following a 24 h period to ensure cell adhesion, the cells were exposed to varying concentrations of berberine (2.5, 5, 10, 20, 40, 80, 160 μM), evodiamine (0.03125, 0.0625, 0.125, 0.25, 0.5, 1, 2 μM), limonin (5, 10, 20, 40, 80, 160, 320 μM), rutaecarpine (2.5, 5, 10, 20, 40, 80, 160 μM), coptisine (2.5, 5, 10, 20, 40, 80, 160 μM), palmatine (5, 10, 20, 40, 80, 160, 320 μM), and epiberberine (5, 10, 20, 40, 80, 160, 320 μM). After 1, 2, and 3 days of treatment, 10 μL of CCK-8 reagent was introduced into each well, and the plates were subjected to an additional 2 h incubation. Thereafter, absorbance measurements at 450 nm were conducted using the SpectraMax iD5 microplate reader. Additionally, the absorbance of the combination of BBE (10 μM berberine + 0.0625 μM evodiamine) was assessed at 450 nm.

### 4.3. Adipocyte Differentiation and Oil Red O Staining

Murine 3T3-L1 fibroblasts were cultured under conditions of 5% CO_2_ and 37 °C, and were seeded in complete growth medium. Upon achieving 100% confluency, the cells were permitted to undergo contact inhibition for an additional two days. Subsequently, 3T3-L1 cells were maintained for 48 h in a medium composed of DMEM enriched with 1% penicillin/streptomycin, 10% fetal bovine serum, 0.5 mM isobutylmethylxanthine, 10 μg/mL insulin, and 1 μM dexamethasone. During this period, cells were treated with the following compounds: berberine (10 μM), rutaecarpine (5 μM), evodiamine (0.0625 μM), palmatine (5 μM), limonin (5 μM), coptisine (5 μM), epiberberine (5 μM), or a combination of berberine (10 μM) and evodiamine (0.0625 μM). Two days later, the cells were further cultivated for an additional 48 h in complete medium supplemented with 10 μg/mL insulin. Finally, the cells were cultured in a completely insulin-free medium for four days until full adipocyte differentiation was achieved, after which they were utilized for subsequent experiments. To conduct ORO staining, the cells underwent two washes with PBS, following the removal of the medium. Subsequently, the cells were immobilized with ORO fixative for 30 min and then rinsed twice with deionized water. The cells were incubated in 60% isopropanol for five minutes, after which they were subsequently stained with ORO solution for 20 min. Poststaining, the cells underwent five washes with deionized water and were examined under a light microscope. Stained lipid droplets were then extracted with 100% isopropanol, and then the absorbance was quantified at 540 nm using a microplate reader.

### 4.4. Animals and Experimental Design

Male C57BL/6J mice, provided by SPF Biotechnology Co., Ltd. (Beijing, China; Certificate No. SCXK-JING 2019-0010), weighed 15–17 g and were 4 weeks old. These animals were provided with unrestricted access to food and water and were housed under standardized environmental conditions, which included a 12 h light–dark cycle, humidity levels of 55 ± 10%, and a temperature of 22 ± 2 °C. Authorization for all animal experimental procedures was granted by the Ethics Committee of Chengdu University of Traditional Chinese Medicine (Approval No. 2021DL-001; approved on: 11 March 2021). These procedures adhered strictly to the Guidelines for the Care and Use of Laboratory Animals set forth by the Ministry of Science and Technology of China.

After seven days of acclimatization, the mice were allocated into seven groups: normal diet group (NCD), high-fat diet group (HFD), 33 mg/kg berberine group (BER), 8 mg/kg evodiamine group (EVO), 33 mg/kg berberine + 8 mg/kg evodiamine group (BEL), 66 mg/kg berberine + 16 mg/kg evodiamine group (BEH), and 1.5 mg/kg atorvastatin calcium group (ATO). The NCD group was fed a standard chow diet (10% fat; D12450J, Research Diet), whereas the remaining groups were provided with a high-fat diet (60% fat; D12492, Research Diets). In addition to their respective diets, the NCD and HFD groups were administered an equivalent volume of vehicle (0.9% sodium chloride) via gavage, while the treatment groups received the corresponding doses of berberine, evodiamine, or atorvastatin calcium. All gavage administrations were performed once daily for 10 consecutive weeks at a volume of 0.1 mL per 10 g of body weight, with the gavage volume adjusted weekly according to the animals’ weight. The mice were housed in clean cages, and oral gavage was performed daily. Body weight, body length, and food intake were monitored weekly. Mice were considered successfully obese if their body weight exceeded the average weight of the NCD group by 20%. The Lee’s index was calculated using the formula: [body weight (g)]^(1/3) × 1000/[body length (cm)]. Upon the conclusion of the experiment, the mice were humanely euthanized under anesthesia, and blood samples were collected. The inguinal, epididymal, scapular brown, and liver tissues were dissected, weighed, and processed for further analysis. Adipose tissue (iWAT, eWAT, and BAT) and liver indices were calculated to assess relative organ mass. These indices were defined as the ratio of tissue weight (g) to total body weight (g) at sacrifice and were calculated using the following formula: Tissue index = [Tissue weight (g)/Body weight (g)] × 100%. The tissue segment was secured in 4% paraformaldehyde, whereas the remaining portion was quickly frozen in liquid nitrogen and preserved at −80 °C.

### 4.5. Analysis of Lipid Profile and Glycemic Indicators

Serum concentrations of TG, AST, LDL-C, TC, ALT, GLU, and HDL-C were quantified using biochemical assay kits (Mindray Bio-medical Electronics Co., Shenzhen, China). The measurements were performed using an automated biochemical analyzer (Mindray Bio-medical Electronics Company, Shenzhen, China). The homeostasis model assessment of insulin resistance (HOMA-IR) was determined by the formula: [fasting blood glucose (mmol/L) × fasting insulin (μU/mL)]/22.5. For the oral glucose tolerance test (OGTT), the mice were fasted for 12 h, after which baseline blood glucose concentrations were determined. A glucose solution of 2 g/kg body weight was administered to the mice via gavage. Blood glucose concentrations were measured at 0, 15, 30, 60, 90, and 120 min postadministration using the Accu-Chek Performa glucose meter (GA-3, Sinocare, Changsha, China). To evaluate glucose tolerance, blood glucose profiles were generated, and the area under the curve (AUC) was determined.

### 4.6. Detection of BBE by ELISA

Serum levels of adiponectin, leptin, and insulin were measured using ELISA kits (ZC-39014W, ZC-38677W, ZC-38920W, respectively; ZCIBIO Technology Co., Ltd., Shanghai, China) following the manufacturer’s guidelines. Optical density (OD) was assessed at 450 nm utilizing a microplate reader (SpectraMax iD5, Molecular Devices, San José, CA, USA). The concentrations of adiponectin, leptin, and insulin were determined based on the standard curves provided with each kit.

### 4.7. JC-1 Flow Cytometry Analysis

The JC-1 Mitochondrial Membrane Potential Assay Kit (E-CK-A301, Elabscience, Wuhan, China) was employed to evaluate mitochondrial membrane potential following the manufacturer’s protocol. Inguinal white adipose tissue was dissociated into a single-cell suspension, and the JC-1 working solution was used to suspend the cells, which were then incubated at 37 °C for 20 min. Following incubation, the cells were rinsed with precooled 1 × JC-1 Assay Buffer and resuspended for analysis using flow cytometry (488 nm laser, FITC and PE channels). The ratio of red fluorescence (aggregates) to green fluorescence (monomers) was used to assess mitochondrial membrane potential changes.

### 4.8. Histological and Immunofluorescence Analysis

Inguinal, epididymal, and scapular brown adipose tissues, as well as liver, were fixed in 4% paraformaldehyde for two days. Fixed tissues were subjected to dehydration, followed by paraffin infiltration, and then sectioned at a thickness of 3 μm. Subsequently, H&E staining was conducted, and the sections were analyzed using a Leica Microsystems pathological slide scanner (Shanghai, China). For immunofluorescence staining of inguinal fat tissue, sections embedded in paraffin were deparaffinized, and endogenous peroxidase activity was suppressed. After incubation with a blocking serum, the sections were then incubated overnight at 4 °C with primary antibodies against UCP1 (1:500, 23673-1-AP, Proteintech) and PRDM16 (1:500, bs-19986R, Bioss). Following PBS washing, incubate the sample with the secondary antibody at 37 °C in the absence of light for a duration of 50 min. Upon subsequent washing, the nuclei were counterstained with DAPI (G1012, Servicebio) at room temperature for 10 min. Then, they were examined under a fluorescence microscope, and images were captured. The mean fluorescence intensity was quantified using ImageJ software (version 1.52a; Bethesda, MD, USA).

### 4.9. Quantitative Reverse Transcription Polymerase Chain Reaction (qRT-PCR) Examination

Total RNA was isolated from 3T3-L1 cells and mouse inguinal fat tissue employing RNAiso Plus (No. 9108, Takara Bio Inc., Tokyo, Japan). The experiment was performed using the PrimeScript FAST RT reagent Kit with gDNA Eraser (RR092A, Takara Bio Inc., Tokyo, Japan) to synthesize complementary DNA (cDNA) from 1 μg of total RNA. Utilizing the TB Green Premix Ex Taq II (CN830A, Takara Bio Inc., Tokyo, Japan), qRT-PCR was carried out on the Bioer qRT-PCR system provided by Hangzhou Bioer Technology Co., Ltd., Hangzhou, China. The thermal cycling protocol begins with a 30 s denaturation at 95 °C, followed by 40 cycles consisting of 5 s of denaturation at 95 °C and 10 s of annealing/extension at 60 °C. Normalization of target gene relative mRNA expression levels was performed against the housekeeping gene *Gapdh*, followed by quantification via the 2^−ΔΔCT^ approach. Table 1 outlines the primer sequences employed for the qRT-PCR analysis.

### 4.10. Western Blot Analysis

RIPA lysis buffer was applied to the inguinal adipose tissue and 3T3-L1 cells from mice, and the samples were homogenized on ice. The lysates were subjected to centrifugation at 13,000× *g* for 10 min at 4 °C. The resultant supernatants were harvested, and protein concentrations were measured using a BCA Protein Assay Kit (Boster Biological Technology Co., Ltd., Pleasanton, CA, USA). A total of 20 μg of protein samples were separated by SDS-PAGE and blotted onto polyvinylidene difluoride (PVDF) membranes. The PVDF membranes were incubated with 5% non-fat milk at room temperature for 2 h. Subsequently, the membranes were incubated with primary antibodies overnight at 4 °C. The primary antibodies used were as follows: anti-UCP1 (1:2000), anti-PGC-1α (1:600), anti-FGF21 (1:1000), and anti-GAPDH (1:60,000). Subsequent to the washing step with TBST, the PVDF membranes were exposed to HRP-conjugated secondary antibodies (1:10,000 dilution) at room temperature for a duration of 1 h. Subsequently, the blots were visualized using an ultra-sensitive chemiluminescent detection kit. The imaging was performed with an eBlot Touch Imager (Shanghai, China), with the exposure settings optimized for clarity. Protein bands were captured following adjustment of the exposure parameters. Finally, the quantification of the Western blot results was conducted using ImageJ software.

### 4.11. In-Silico Analysis

The three-dimensional crystal structures of UCP1 (PDB: 8HBV), PGC-1α (PDB: 8BF1), PRDM16 (PDB: 2N1I), CIDEA (PDB: 2EEL), FGF21 (PDB: 6M6E), and FGFR1c (PDB: 5EW8) were retrieved from the Protein Data Bank (PDB). Additionally, the three-dimensional structures of berberine hydrochloride (Compound CID: 12456) and evodiamine (Compound CID: 442088) were retrieved from the PubChem database. All protein structures were processed by eliminating water molecules, introducing polar hydrogen atoms, and assigning suitable charges using AutoDockTools 1.5.6. The ligands (berberine and evodiamine) were energy-minimized and prepared for docking by setting their rotatable bonds. The molecular docking simulations were performed using AutoDock Vina to conduct semi-flexible docking between the small molecule ligands and the macromolecular receptors [89]. The search space for docking was delineated by defining the grid box dimensions according to the active site of each protein. Following the docking process, the conformations exhibiting the lowest binding energies were chosen for subsequent analysis. The binding affinities were calculated, and the conformations with the best binding energies were visualized using PyMOL v1.7.2.1 (New York City, NY, USA) to illustrate the detailed interactions between the ligands and the receptor proteins.

### 4.12. Statistical Analysis Methods

All data were evaluated using GraphPad Prism 9.0 (GraphPad Prism software, San Diego, CA, USA) and are presented as the mean ± SEM for in vitro and in vivo assays, respectively. Statistical significance was evaluated using one-way and two-way analysis of variance (ANOVA), followed by Fisher’s LSD test. *p* values below 0.05 were deemed statistically significant. 

## 5. Conclusions

This study demonstrates that the combination of berberine and evodiamine effectively combats obesity by activating the FGF21/PGC-1α signaling pathway and promoting browning. In vitro experiments showed that berberine and evodiamine significantly inhibited lipid accumulation and upregulated key markers of brown fat and thermogenesis. In vivo, BBE-treated C57BL/6J mice on a high-fat diet exhibited reduced body weight, improved dyslipidemia, alleviated hepatic steatosis, enhanced glucose tolerance, and favorable changes in adipocytokine levels. Molecular docking and visualization analyses revealed that BBE enhanced mitochondrial activity in iWAT and upregulated browning-related genes and proteins. These findings suggest that BBE increases energy expenditure by promoting browning and enhancing mitochondrial function and thermogenesis via the FGF21/PGC-1α pathway. This novel strategy holds significant potential for controlling obesity and related metabolic disorders.

## Figures and Tables

**Table 1:** Primers sequences.

Primers	Sequence (5′ to 3′)
PGC-1α-F	ATGTGTCGCCTTCTTGCTCTT
PGC-1α-R	GGACCTTGATCTTGACCTGGAA
UCP1-F	AGGCTTCCAGTACCATTAGGT
UCP1-R	CTGAGTGAGGCAAAGCTGATTT
PRDM16-F	GCCGTTCAAGTGCCATCTGT
PRDM16-R	CCTCGTGTTCGTGCTTCTTCA
CIDEA-F	TCCTCGGCTGTCTCAATGTCA
CIDEA-R	GGATGGCTGCTCTTCTGTATCG
DIO2-F	GTGGCTGACTTCCTGTTGGTAT
DIO2-R	GCACATCGGTCCTCTTGGTT
FGF21-F	AGCACACCGCAGTCCAGAA
FGF21-R	GTCCTCCAGCAGCAGTTCTCT
FGFR1c-F	GATGACCTCACCGCTCTACCT
FGFR1c-R	TCTGGCTATGGAAGTCGCTCTT
GAPDH-F	CAGTGGCAAAGTGGAGATTGTTG
GAPDH-R	TCGCTCCTGGAAGATGGTGAT

## Data Availability

Data will be made available on request.

## Abbreviations

AUC	area under the curve	HOMA	homeostasis model assessment
AT	adipose tissue	IBMX	3-Isobutyl-1-methylxanthine
CCK8	Cell Counting Kit-8	OGTT	oral glucose tolerance test
DAPI	4ʹ,6-diamidino-2-phenylindole dihydrochloride	qRT-PCR	quantitative reverse transcription polymerase chain reaction
DMEM	Dulbecco’s modified Eagle’s medium	RIPA	radio immunoprecipitation assay
FBS	fetal bovine serum	RT	room temperature
H&E	hematoxylin and eosin	iWAT	inguinal white adipose tissue
HDL-C	high-density lipoprotein cholesterol	eWAT	epididymal white adipose tissue
LDL-C	low-density lipoprotein cholesterol	BAT	interscapular brown adipose tissue
TC	total cholesterol	UCP1	uncoupling protein 1
TG	triglyceride	DMSO	dimethyl sulfoxide
SDS-PAGE	SDS-polyacrylamide gel electrophoresis	PBS	phosphate-buffered saline
PVDF	polyvinylidene difluoride	WHO	World Health Organization
OD	optical density	WAT	white adipose tissue
NAFLD	non-alcoholic fatty liver disease	TCM	traditional Chinese medicine
BER	berberine hydrochloride	ALT	alanine aminotransferase
EVO	evodiamine	AST	aspartate aminotransferase
BBE	a combined berberine and evodiamine treatment	GLU	glucose
PAL	palmatine hydrochloride	FBS	fetal bovine serum
EPI	epiberberine	P/S	penicillin-streptomycin liquid
RUT	rutaecarpine	DEX	dexamethasone
HFD	high-fat diet	NCD	normal chow diet
COP	coptisine chloride	LIM	limonin
PGC-1α	peroxisome proliferator-activated receptor gamma coactivator 1-alpha	ATO	atorvastatin Calcium
PRDM16	PRdomain-containing16	CIDEA	Cell Death-Inducing DFF45-Like Effector A
